# An image-based high-content screening for compounds targeting *Toxoplasma gondii* repurposed inhibitors effective against the malaria parasite *Plasmodium falciparum*

**DOI:** 10.3389/fcimb.2023.1102551

**Published:** 2023-03-03

**Authors:** Ariane Honfozo, Rodrigue Houngue, Alexandre Vandeputte, Sébastien Dechavanne, Odilon Nouatin, Ménonvè Cynthia Atindehou, Lucie Ayi Fanou, Achille Massougbodji, Célia Dechavanne, Priscille Brodin, Stanislas Tomavo

**Affiliations:** ^1^ Université Paris Saclay, CNRS UMR 9198-CEA, Institute for Integrative Biology of the Cell (I2BC), Gif sur Yvette, France; ^2^ Université de Lille, CNRS UMR 9017, INSERM U 1019, Institut Pasteur de Lille, US 41 − UAR 2014 − PLBS, CIIL − Center for Immunity and Infection of Lille, Lille, France; ^3^ Université de Paris Cité, IRD, MERIT, Paris, France; ^4^ CERPAGE, Cotonou, Benin; ^5^ Institut de Recherche Clinique du Bénin, Abomey-Calavi, Benin; ^6^ Laboratoire de Biochimie et de Biologie Moléculaire, FAST/UAC, Cotonou, Benin

**Keywords:** *Toxoplasma gondii*, TgSORT-GFP, ROP1-mCherry, image-based analysis, high-content screening, repurposing drugs, malaria parasite

## Abstract

*Apicomplexa* phylum includes numerous obligate intracellular protozoan parasites that are life threatening for humans and animals. In this context, *Plasmodium falciparum* and *Toxoplasma gondii* are of particular interest, as they are responsible for malaria and toxoplasmosis, respectively, for which efficient vaccines are presently lacking and therapies need to be improved. Apicomplexan parasites have a highly polarized morphology, with their apical end containing specific secretory organelles named rhoptries and micronemes, which depend on the unique receptor and transporter sortilin TgSORT for their biogenesis. In the present study, we took advantage of the subcellular polarity of the parasite to engineer a clonal transgenic *Toxoplasma* line that expresses simultaneously the green fluorescent protein TgSORT-GFP in the post-Golgi-endosome-like compartment and the red fluorescent protein rhoptry ROP1-mCherry near the apical end. We utilized this fluorescent transgenic *T. gondii* to develop a miniaturized image-based phenotype assay coupled to an automated image analysis. By applying this methodology to 1,120 compounds, we identified 12 that are capable of disrupting the *T. gondii* morphology and inhibiting intracellular replication. Analysis of the selected compounds confirmed that all 12 are kinase inhibitors and intramembrane pumps, with some exhibiting potent activity against *Plasmodium falciparum*. Our findings highlight the advantage of comparative and targeted phenotypic analysis involving two related parasite species as a means of identifying molecules with a conserved mode of action.

## Introduction

The phylum *Apicomplexa* includes about 5,000 intracellular protozoan parasites that infect humans and animals, such as *Plasmodium falciparum* (the causative agent of human malaria), *Toxoplasma gondii* (an important opportunistic pathogen associated with AIDS and congenital birth defects. This phylum also contains harmful infections in animals, *Eimeria* (a causative agent of deadly coccidiosis in poultry) and *Cryptosporidium* (an opportunistic intestinal pathogen causing severe diarrhea). Malaria is an ancient vector-borne infectious disease primarily occurring in developing countries that is responsible for about 600,000 deaths per year, most of which occur in children (WHO, 2021). On the other hand, toxoplasmosis is less deadly, but *in utero* exposure can lead to devastating outcomes, including congenital malformations such as blindness, lack of normal development of intellectual capacities and hydrocephaly in newborns. In addition, in HIV-positive individuals, it causes severe opportunistic infections, which is of major public health concern as it results in physical and psychological disabilities (Montoya and Liesenfeld, 2004; Halonen and Weiss, 2013; Milne et al., 2020).

Toxoplasmosis is presently treated with a combination of drugs, such as pyrimethamine and sulfadiazine, and the affected individuals are typically prescribed folinic acid to prevent suppression of bone marrow formation *via* pyrimethamine (Hill and Dubey, 2002). However, these drugs have severe side effects, such as neutropenia, leucopenia, severe reduction in platelet count, and hypersensitivity (Porter and Sande, 1992; Rajapakse et al., 2013). Therefore, clindamycin may be used as an alternative. When patients are unable to tolerate either sulfonamides or clindamycin, atovaquone can be prescribed (Dunay et al., 2018), whereas spiramycin is recommended for pregnant women due to its bioavailability in the placenta. In seropositive AIDS patients, trimethoprim in combination with sulfamethoxazole has been shown to prevent cerebral toxoplasmosis. Nonetheless, most of these drugs are poorly tolerated and their long-term use results in cytotoxicity, or fails to deliver expected outcomes. Likewise, malaria is currently treated *via* an artemisinin-based combination therapy, which is increasingly ineffective as a result of the growing resistance of the malaria parasite *P. falciparum* to the available drugs (Balikagala et al., 2021).

*Apicomplexa* are complex single-celled eukaryotes containing classical nucleus, mitochondrion, endoplasmic reticulum and Golgi, as well as specific secretory organelles—rhoptries and micronemes—that are located at the apical end of the parasites, whose contents are required for host cell attachment, invasion and virulence. Extant research indicates that many rhoptry proteins are kinases or pseudokinases capable of defining virulence factors that can be secreted in different compartments of the infected host cells, including the nucleus. For example, ROP16 can phosphorylate STAT3/6 and thus control the transcription level of numerous genes involved in the host’s immune response (Saeij et al., 2006; Taylor et al., 2006; Saeij et al., 2007; Yamamoto et al., 2009; Ong et al., 2010; Butcher et al., 2011). In contrast, the microneme MIC proteins are defined as attachment factors required by the parasite to recognize the receptors on the host cell surface during entry (Huynh et al., 2003; Huynh and Carruthers, 2006). In addition, ROP and MIC proteins can act synergistically to form the moving junction necessary for the host cell invasion (Alexander et al., 2005; Lamarque et al., 2011). Available evidence further indicates that apicomplexan parasites, including *T. gondii*, are 10 µm long eukaryotic organisms with a highly polarized secretory pathway. Several authors have also noted that proteins and probably lipids destined for the apical secretory organelles (i.e., rhoptries and micronemes) are synthesized in the ER and move to the Golgi apparatus before reaching the post-Golgi and endosomal-like compartment (Pelletier et al., 2002; He, 2007; Tomavo et al., 2013). ROP and MIC proteins are also synthesized as pro-proteins that are cleaved at the N-terminus during maturation, leading to properly folded and functionally active proteins (Bradley and Boothroyd, 2001; Harper et al., 2006; Brydges et al., 2008). In our earlier work, we demonstrated that the transport and maturation of ROP and MIC proteins required the presence of an essential sortilin homologue, which we denoted as *T. gondii* sortilin–like receptor (TgSORT) (Sloves et al., 2012). In addition, we showed that the rhoptry and microneme biogenesis depends on TgSORT. These findings were subsequently confirmed by Sangaré et al. (2016) who reported that the luminal domain of the receptor binds rhoptry and microneme proteins, while the cytosolic tail recruits partners to enable anterograde and retrograde receptor transport. This evidence indicates that TgSORT is a key receptor for the biogenesis of secretory organelles in *T gondii* as well as *P. falciparum*, and its homologue PfSortilin in malaria was later found to control transport of proteins to form rhoptries (Hallée et al., 2018a, 2018b). Because the homologues of TgSortilin are present in all apicomplexan parasites whose genome has been sequenced (VEuPathDB), we hypothesize that this receptor is a key factor that allows apicomplexan parasites to build the complex apical structure composed of a functional conoid containing rhoptries and micronemes. We further posit that protein trafficking is a critical parameter in parasite multiplication and dissemination. Therefore, identifying molecules that target this process could enrich the therapeutic arsenal. In the present study, we exploit the *T. gondii* cell polarity to engineer a clonal transgenic *Toxoplasma* line expressing TgSORT-GFP (green fluorescence) located in the post-Golgi/endosome-like compartment (ELC) and an apically located rhoptry ROP1-mCherry to develop an image-based high-content screening approach. The proposed method allowed us to identify twelve compounds that disrupt the parasite polarity and block intracellular replication of *T. gondii.* We further showed that four of these compounds were also potent inhibitors of *P. falciparum*.

## Materials and methods

### Parasite strains and reagents

The following *T. gondii* strains were used in this work: RH strain, RHΔKU80, and RH containing Lac Z (clone 2F1) expressing β-galactosidase, which were kindly provided by Prof. Vern Carruthers (University of Michigan, USA) and *P. falciparum* 3D7 strain procured from the Institut de Recherche et de Développement (IRD-Bénin). The following reagents were also used: DAPI, (D9542, Sigma-Aldrich), crystal violet (C0775, Sigma Aldrich), Kit RAL 555 (RAL Diagnostics, Labelians, Belgium), 5’-fluorodeoxyuridine (5’FUDR, Merck), pyrimethamine (Merck), mycophenolic acid (MPA, Calbiochem), Xanthine (XAN, Merck), chlorophenol red-β-D-galactopyranoside (CPRG, Merck), Hoechst-Thiazole Orange (Sigma Aldrich) and Tocriscreen Total library (Cat. No. 2884, Tocris Biotechne).

### Intracellular growth of *T. gondii*

The tachyzoites of *T. gondii* strains used in the present study were routinely cultured on a human fibroblast foreskin (HFF) monolayer in Dulbecco’s Modified Eagles Medium (DMEM, PAN Biotech, Dutscher, France) supplemented with heat-inactivated 10% fetal bovine serum (PAN Biotech, Dutscher, France), 2 mM of glutamine and 50 µg/mL of penicillin/streptomycin (PAN Biotech, Dutscher, France). After three hours, the infected cells were washed with culture medium and the intracellular parasites were allowed to grow at 37°C for 2−3 days to ensure complete lysis. Freshly lysed tachyzoites were purified using 0.33 µm filter (Millipore) to remove cell debris before counting, and the obtained parasites were used for transfection or for drug assays.

### Plasmid constructs and generation of transgenic *T. gondii*

Transgenic ROP1-mCherry parasites were obtained *via* a knock-in strategy using a 2283 bp DNA fragment as well as KI-ROP1 forward primer (TAC TTC CAA TCC AAT TTA ATG CTG GGC TCG CAC CAA TAG CAC) and KI-ROP1 reverse primer (TCC TCC ACT TCC AAT TTT AGC TTG CGA TCC ATC ATC CTG CTC). This DNA fragment was cloned into the pLIC-mCherry-HXGPRT (hypoxanthine-xanthine-guanine phosphoribosyltransferase selectable marker) plasmid (Huynh and Carruthers, 2009), and was linearized using BstBI restriction enzyme. Tachyzoites (5×10^6^ parasites) of the RHΔKu80 strain were transfected with 25 µg of linearized plasmids. After two selections with 25 µg/mL MPA and 50 µg/mL XAN, stable parasites were cloned and positive clones were screened by indirect immunofluorescence assay (IFA), whereby three individual clones were selected for further confocal imaging. One of the positive clones was then knocked out for the uracil phosphoribosyl transferase (UPRT) gene using a second plasmid containing TgSORT-GFP as previously described Sloves et al. (2015), whereby the plasmid containing the HA tag was replaced by GFP. All aforementioned plasmids were checked for accuracy by full DNA sequencing before use. Transgenic tachyzoites (5x10^6^) expressing rhoptry ROP1-mCherry were transfected with 25 µg of linearized promSORT-TgSORT-GFP plasmid and were subsequently transferred to the monolayer HFF for 4h, and were washed with culture medium. This was followed by a 24-hour growth, after which 5 µM of 5’FUDR was added. After three days, selection was repeated twice before cloning the emerging resistant parasites. After screening by IFA, one positive clone was selected for drug screening using the Tocris library as described below.

### *Toxoplasma* strains and cell infection for the miniaturized high-throughput assay

*T. gondii* RH-ΔKu80 expressing ROP1-mCherry/TgSORT-GFP (clone D6) tachyzoites were maintained by growth on HFF cell monolayers as previously described, whereby HFF cells at 12 sub-cultured dilution cycles kept in liquid nitrogen prior to their use, and were cultivated in complete DMEM medium after thawing in a 75-cm^2^ flask. Three days later, the cells were trypsinized, resuspended to complete the DMEM medium, and transferred to a 175-cm^2^ flask until confluency had been attained. Prior to infection, the culture medium was removed and HFF cells were washed twice with sterile phosphate buffered saline (PBS) after which 3 mL of trypsin-EDTA was added and the sample was incubated for 4 minutes at 37°C. After verifying cell detachment by microscope, 25 mL of growth medium was added and HFF cells were then counted using a TALI Cytometer (Invitrogen) and were infected at MOI (multiplicity of infection) 2.5 with freshly lysed sourced from a 25-cm^2^ flask containing infected HFF cells and filtered through 3 µM membrane (polycarbonate, Whatman). After parasite counting using Malassez hematocytometer, the infected cells were grown for 4 hours at 37°C, and the HFF cell monolayer was washed with PBS, trypsinized, and resuspended with complete culture medium before counting. The contents were diluted to obtain a final concentration of 3.25×10^5^ cells per mL and these infected cells were immediately used for drug library screening.

### Compound screening and image acquisition

Tested compounds were obtained from the Tocriscreen Total library (Cat. No. 2884, Tocris), which contains 1,120 biologically active compounds solubilized in pure DMSO at 10 mM. Compound solutions were stored in specific plates to allow Acoustic Droplet Emission transfers (**Supplementary Figure 1**), which were performed using an Echo550^®^ (Labcyte), allowing us to dispense 40 nL of each compound to reach a 10 µM concentration in the 40 µL volume used for the test, with DMSO serving as negative control (1% final concentration in the assay). Initially, assays were performed using pyrimethamine (Pyr) as a positive control (at 0.6 µM and 10 µM concentrations), as this drug is known to efficiently and rapidly kill *T. gondii* at very low concentrations. After the initial identification, SB 203580 (Cpd1) and SB 208 (Cpd3) were used as positive controls. For compound screening, 40 µL (corresponding to 1.3×10^5^ cells) of infected-cell suspension was dispensed in each well of 384-well plates containing compounds, which were prepared and kept for a few minutes at room temperature before use. These plates were incubated at 37°C in 5% CO_2_ and 95% humidity atmosphere for 24 hours before further processing.

### Sample fixation and staining

After 24-hour of incubation, infected cells were subjected to drug effects, and the cell culture media and drugs were removed, after which 20 µL of PBS was added to each well using a washer-dispenser device (El406^®^ from Biotek), whereby 20 µL of 10% formalin solution was placed on top of the PBS. Plates were kept at room temperature for 20 minutes and were washed once with PBS before staining with DAPI prepared at a 2 µg/mL concentration in PBS containing 0.1% of Triton X-100, 100 mM of glycine and 5% fetal bovine serum (FBS). After incubating at room temperature for 20 minutes, plates were washed with 50 µL of PBS, and 40 µL of PBS containing 1% FBS was added to each well, after which the plate was sealed and stored at 4°C until required for image acquisition, performed using an InCell Analyzer 6000^®^ (GE Healthcare). For this purpose, six fields per well were acquired from the A1 well and were placed in the P24 well in a horizontal serpentine mode of acquisition with the 60× objective and the exposure parameters were indicated in **Table 1**.

**Table 1:** Exposure parameters of image acquisition using the InCell Analyzer.

Laser wavelength (in nm)	Emission Filter (in nm)	Exposition time (in ms)
405	455	200
488	525	500
561	605	200

Images from all three channels (hereafter denoted as blue, green and red, respectively) were captured in confocal mode, using the closed aperture option in order to reduce background noise.

### Image analysis

Images captured by the InCell Analyzer 6000^®^ were analyzed using Columbus software. Both cell- and parasite-based analyses were performed, and were supplemented by vacuole structure and organization assessments in certain cases. First, a local maxima detection algorithm was applied to the DAPI channel to detect the nuclei, which were then thresholded by size, roundness and DAPI mean intensity to avoid any false detection (as parasite vacuoles could be misidentified as nuclei). Next, cytosols were detected based on the DAPI background signal in this region, after which a spot detection on the DAPI channel was processed to detect parasites in the entire cell layer. Based on this parameter, parasite count per field or per HFF cell monolayer was obtained. In parallel, as parasites were gathered in vacuoles, the vacuole population was split into positive and negative groups. Given that positive vacuoles were considered to express the expected mislocalisation phenotype, they were selected using a multiparameter threshold, including the ratio of ROP1-mCherry intensity between the center and the border of the vacuole and the mean mCherry intensity in the vacuole. To be labelled as positive, vacuoles had to fulfill the following two criteria: (1) the ratio of intensity between the center and the border of the vacuole must exceed 1.4, and (2) the mean intensity in the vacuole must be below 500, as explained in detail in **Supplementary Table 2**. The percentage of positive vacuoles was thus used as a parameter that reflects the vacuole disruption phenotype. The data were normalized using Z-score.

### Indirect immunofluorescence using confocal microscopy

Intracellular parasites were fixed with 4% paraformaldehyde prepared in PBS for 30 minutes at room temperature, and were washed three times with PBS before staining with DAPI. Samples were observed with a Zeiss confocal microscope and the obtained images were processed using Image J software. When antibodies were utilized, the samples were permeabilized with 0.2% Triton X-100 prepared in PBS with 100 mM of glycine to block free aldehyde groups, incubations were done for 30 min at room temperature all along the experiment. After blocking with 10% of FBS in PBS containing 0.1% Triton X-100, monoclonal or polyclonal antibodies were added at 1:500 dilutions and incubated at 37°C for 30 min. After three washes, Alexa 488 nm or 560 nm secondary antibodies were added to the same buffer with DAPI and blue Evans, and after incubation at 37°C for 30 min, and three washes with PBS containing 0.1% of Triton X-100, the coverslips were mounted with Mowiol and dried at 37°C before confocal microscopy observations.

### β-galactosidase assay

Purified tachyzoites (2×10^5^ parasites) of the transgenic *T. gondii* RH (clone 2F1) strain were used to infect confluent HFF cell monolayer in 24 cm^2^-well plates for 4 hours and, after washing once with culture medium, the infected cells were incubated with compounds at different concentrations for 48 hours. After recovery by scraping, materials were centrifuged at 5,000 rpm for 10 minutes at 4°C. The pellets were washed once with PBS and lysed for 60 minutes at 50°C in 150 µL of a buffer containing of 1% Triton X-100, 5 mM DTT, 1 mM MgSO_4_ and 100 mM HEPES as described previously (Seeber and Boothroyd, 1996; McFadden et al., 1997). After centrifugation at 10,000 rpm for 5 minutes at 4°C, 50 µL of the supernatant was diluted to 50% with lysis buffer. After adding 100 µL of 2 mM chlorophenol red-β-D-galactopyranoside (CPRG), the resulting mixtures were incubated at 30°C overnight and the optical density was measured at λ = 570 nm.

### Plaque formation

Plaque assays were performed using 24-well plates containing at least 5 day-confluent HFF cells infected with 10^3^ parasites per well in media, with DMSO as negative control, or in DMSO combined with drugs at 25 µM or 50 µM concentration. Nine days post-infection, the controls and infected HFF cells were stained with crystal violet, as previously described (Sloves et al., 2012; Sangaré et al., 2016). Two independent experiments were performed with identical results.

### *In vitro* growth of *P. falciparum*

*P. falciparum* 3D7 strain was used at the Institut de Recherche et de Développement (IRD-Bénin) to obtain parasite cultures from group O+ red blood cells (RBCs) of healthy adult volunteers. RBCs were washed twice in PBS 1X and then in complete malaria culture medium (CMCM) composed of RPMI 1640 (PAN Biotech, Dutscher, France), 0.8% Albumax, 25 mM HEPES, 0.4 mM hypoxanthine, 0.05 mg/mL gentamicin and 2 mM L-glutamine before storage at 4°C. RBCs infected with *P. falciparum* 3D7 were grown at 5% hematocrit at 37°C in an atmosphere comprising 5% CO_2_, 1% O_2_ and 94% N_2_. The culture medium was renewed daily and samples were checked for evidence of parasitemia. Prior to drug testing, the infected RBCs were synchronized with 5% sorbitol for 15 minutes at room temperature, and a second synchronization was performed after 48 hours to eliminate persistent mature forms. Compounds were then tested in 96-well flat-bottom plates at a hematocrit of 5% and an initial ring parasitemia of 1% in 250 µL of CMCM. The following concentrations were tested: 5 µM, 25 µM, 50 µM, 100 µM and 150 µM drugs solubilized by DMSO and prepared in CMCM. The experiments were done in quadruplicate and DMSO was used as the negative control. Chloroquine, a well-known anti-plasmodial drug used at 0.005−150 µM served as the positive control. Plates were incubated for 48 hours at 37°C under the same conditions. Thin smears were created by spreading 6 µL of parasitized RBCs (pRBCs) on a glass slide, which was fixed and stained using the RAL 555 kit before using the AxioCam MRc (color) CCD Rev3 camera of ZEISS microscope at X63 to observe and photograph the RBC smears and process the data using ImageJ software.

### Flow cytometry analysis

Both untreated and drug-treated infected RBCs (2.5 µL) were collected from each well and were incubated at 37°C for 60 minutes in a Hoechst 33258-Thiazole Orange mixture at 0.001 mg/mL and 0.005 mg/mL final concentrations, respectively (Grimberg et al., 2008). Samples were analyzed using BD FACS Canto flow cytometer, whereby FlowJO was used for data analysis and statistical analyses were conducted using GraphPad Prism.

### Statistical analyses

Statistical analyses involved two-way ANOVA followed by Tukey’s multiple comparisons test, which were performed using GraphPad Prism version 8.3.0 (GraphPad Software, San Diego, California USA), with p < 0.05 indicating statistical significance. The results are reported as mean of parasite growth percentage ± SD as a function of the logarithm of inhibitor concentration.

## Results

### Generation of transgenic *T. gondii* TgSORT-GFP/ROP1-mCherry

**Figure 1:**
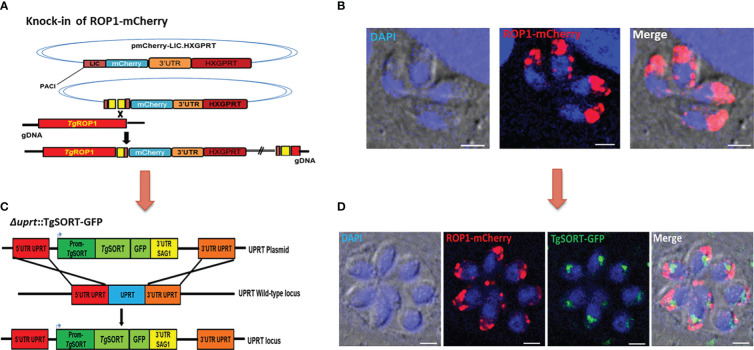
Generation of transgenic *T. gondii* expressing TgSORT-GFP/ROP1-mCherry. **(A)** corresponds to the vector that allows the knock-in of ROP1-mCherry in the parasites. One clone was selected and the red color indicates the ROP1 signal located at the apical end of four intracellular dividing tachyzoites **(B)**. Thus, the vector expressing the TgSORT-GFP was transfected **(C)** and one positive clone (green signal), which replicates into eight intracellular tachyzoites **(D)** for the expression of this receptor in the post-Golgi and endosome-like compartment (ELC) is shown. Bar scale = 3 µm.

With the aim of screening small molecules on a large scale, we created a transgenic *T. gondii* strain that simultaneously expresses ROP1-mCherry (red fluorescent signal) and TgSORT-GFP (green fluorescent signal) as schematically depicted in **Figures 1A**, **C**. For this purpose, we chromosomally appended an encoded mCherry to the rhoptry protein 1 (ROP1) using the plasmid mCherry-LIC-HXGPRT (as explained in the Materials and Methods section and shown in **Figure 1A**). This knock-in strategy was adopted, as it ensures steady-state levels of epitope-tagged protein expression *via* homologous promoters. As indicated in **Figure 1B**, it resulted in a clonal *T. gondii* strain expressing ROP1-mCherry at the apical end of intracellular dividing tachyzoites. Next, we transfected a second vector that expresses TgSORT-GFP, thereby disrupting the uracil phosphoribosyl transferase (UPRT) gene by double homologous recombination and conferring the resistance to 5’FUDR (**Figure 1C**). The UPRT gene was chosen because it is non-essential and its knockout has no phenotypic consequences for the parasite, allowing it to salvage uracil from the infected host cells (Fox and Bzik, 2002).

As shown in **Figure 1D**, we also selected a positive clone for TgSORT-GFP (green signal) in the background of the transgenic *T. gondii* already expressing ROP1-mCherry (red signal). These intracellular parasites dividing transgenic tachyzoites of *T. gondii* simultaneously exhibited TgSORT-GFP in the post-Golgi/endosomal-like compartment and ROP1-mCherry at the apical end of the parasites. It is worth noting that these transgenic parasites showed identical rate of multiplication to the parental or wild type parasites growing inside the human foreskin fibroblasts under our experimental *in vitro* conditions.

### Image-based miniaturized assay

**Figure 2:**
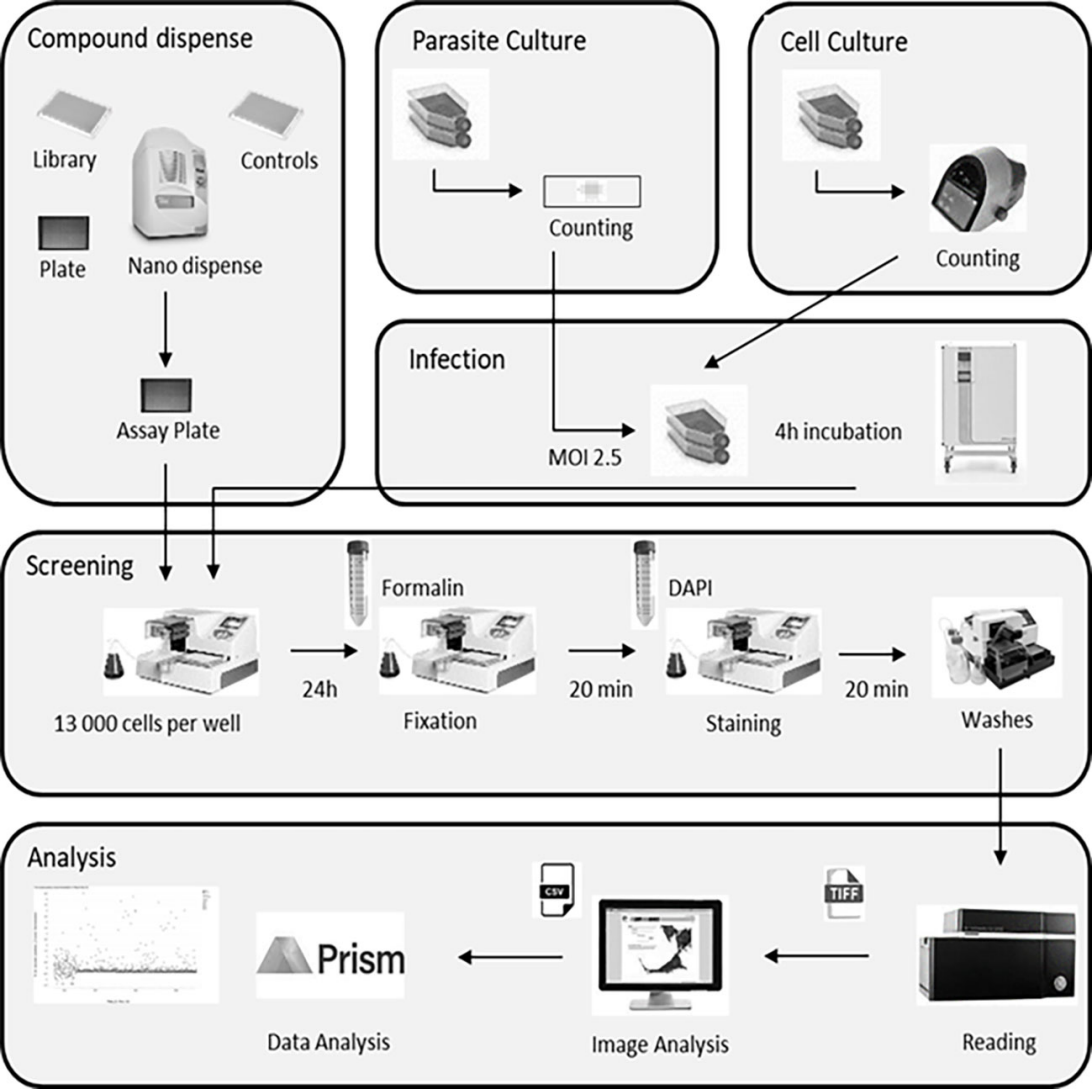
Assay workflow showing all steps of the library screening protocol, namely cell culture, parasite infection, sample processing and labeling, and image acquisition followed by automated image analysis. MOI, multiplicity of infection.

**Figure 3:**
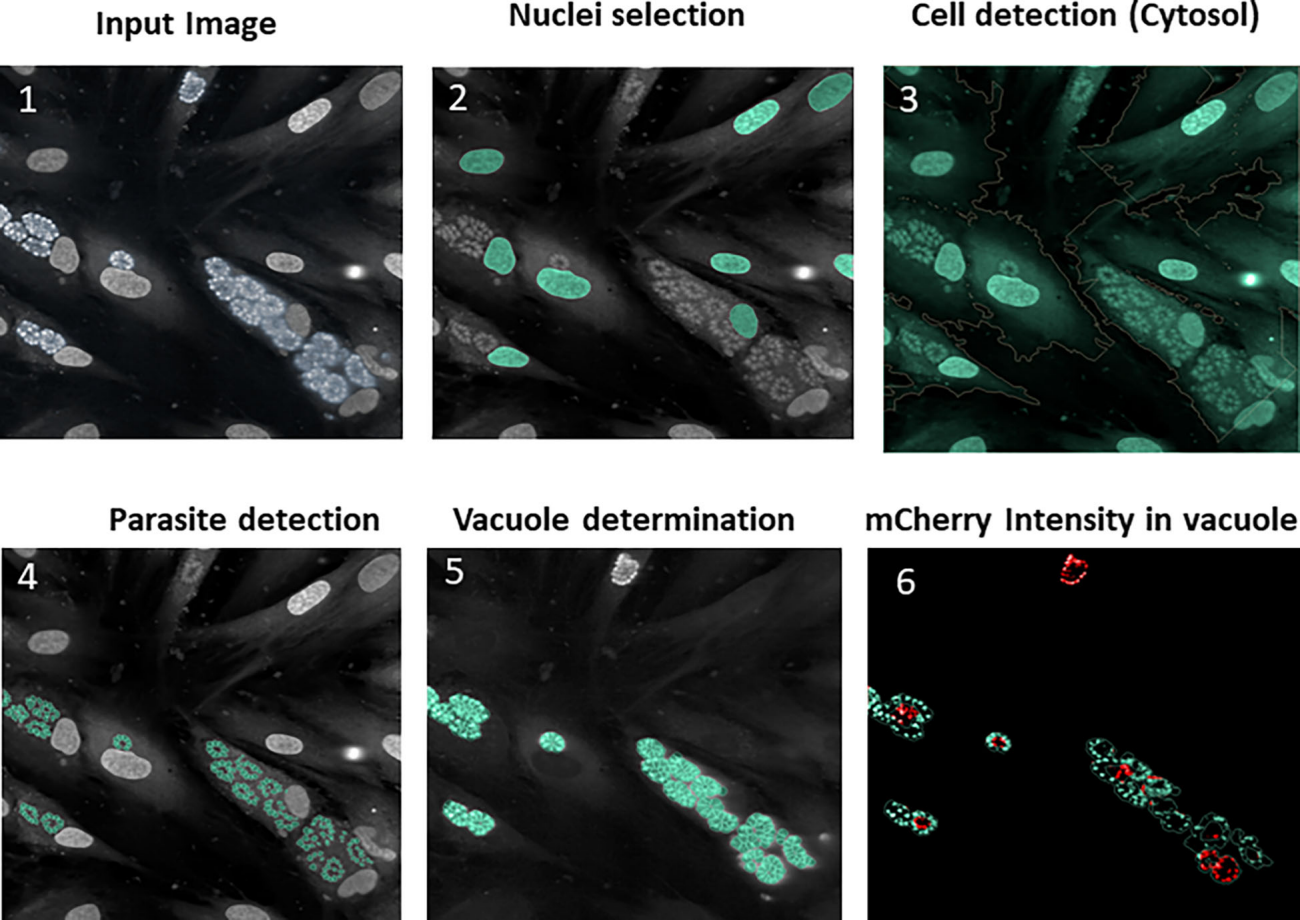
Typical images and image analysis workflow (described in detail in the Columbus analysis script): (1) Raw image acquisition from the reader; (2) Segmentation on the blue channel to detect the HFF nuclei (stained with DAPI reagent), followed by false detection removal based on the intensity/morphology properties in order to retain true nuclei only; (3) HFF cytosol delimitation around nuclei using predefined algorithm based on the use of the DAPI channel, which also stains cytosol (producing a signal of lower intensity); 4) Parasite detection within the cytosol region allowing the DAPI-labeled plots to be segmented into parasite nuclei; (5) Vacuole determination by the total proximal parasites nuclei area (as parasites grow in the vacuole the nucleus area can be mathematically expanded around them); (6) mCherry intensity, enabling determination of disrupted vacuoles. In order for a vacuole to be considered as positive, the mean of the mCherry fluorescence intensity should not be too high and the ratio of mCherry intensity between the vacuole center and the periphery must exceed 1.4.

For the assay, HFF cells were first infected with a pre-culture of *T. gondii* expressing both TgSORT-GFP and ROP1-mCherry, as shown in **Figure 2** depicting assay workflow. The infected cells were subsequently harvested onto a compound containing 384-well plates and were incubated for 24 hours, after which the samples were fixed and stained with nuclear label-DAPI. Next, confocal images were acquired using an automated microscope and well-based analysis was performed using Columbus software (**Supplementary Table 1**; **Figure 3**). To establish the assay, the number of HFF cells, the multiplicity of infection (MOI), and the DMSO impact have to be determined to ensure a homogenous *T. gondii-*infected HFF layer in each well at read-out (**Supplementary Figure 2**). We have chosen pyrimethamine (Pyr) for this purpose, as this well-known drug is frequently used in toxoplasmosis treatment (Hill and Dubey, 2002; Halonen and Weiss, 2013; Dunay et al., 2018) and is an effective parasite growth inhibitor in assays (**Supplementary Figure 2**). A dose response of Pyr was determined and the number of parasites per field was quantified. For DMSO, 18 parasites were noted on average per field, declining to 5 parasites in the presence of 0.6 µM Pyr.

### Small molecule screen for identifying known compounds that inhibit *T. gondii* intracellular growth

**Figure 4:**
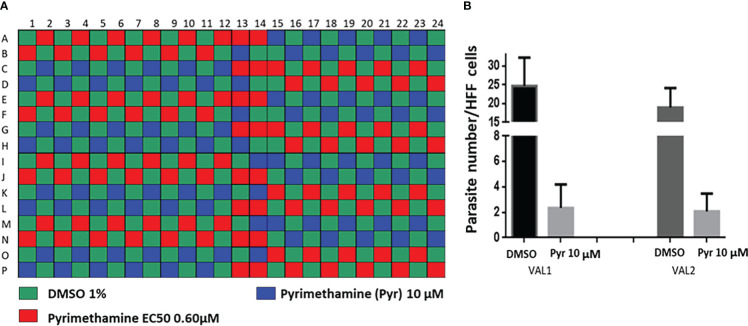
Pre-screen optimization using pyrimethamine, the well-known anti-*Toxoplasma* drug as control. **(A)** Checker-board layout; **(B)** Determination of the parasite number per HFF cells for DMSO and Pyr at 10-µM concentration. VAL1 and VAL2 correspond to the two analyzed plates.

**Figure 5:**
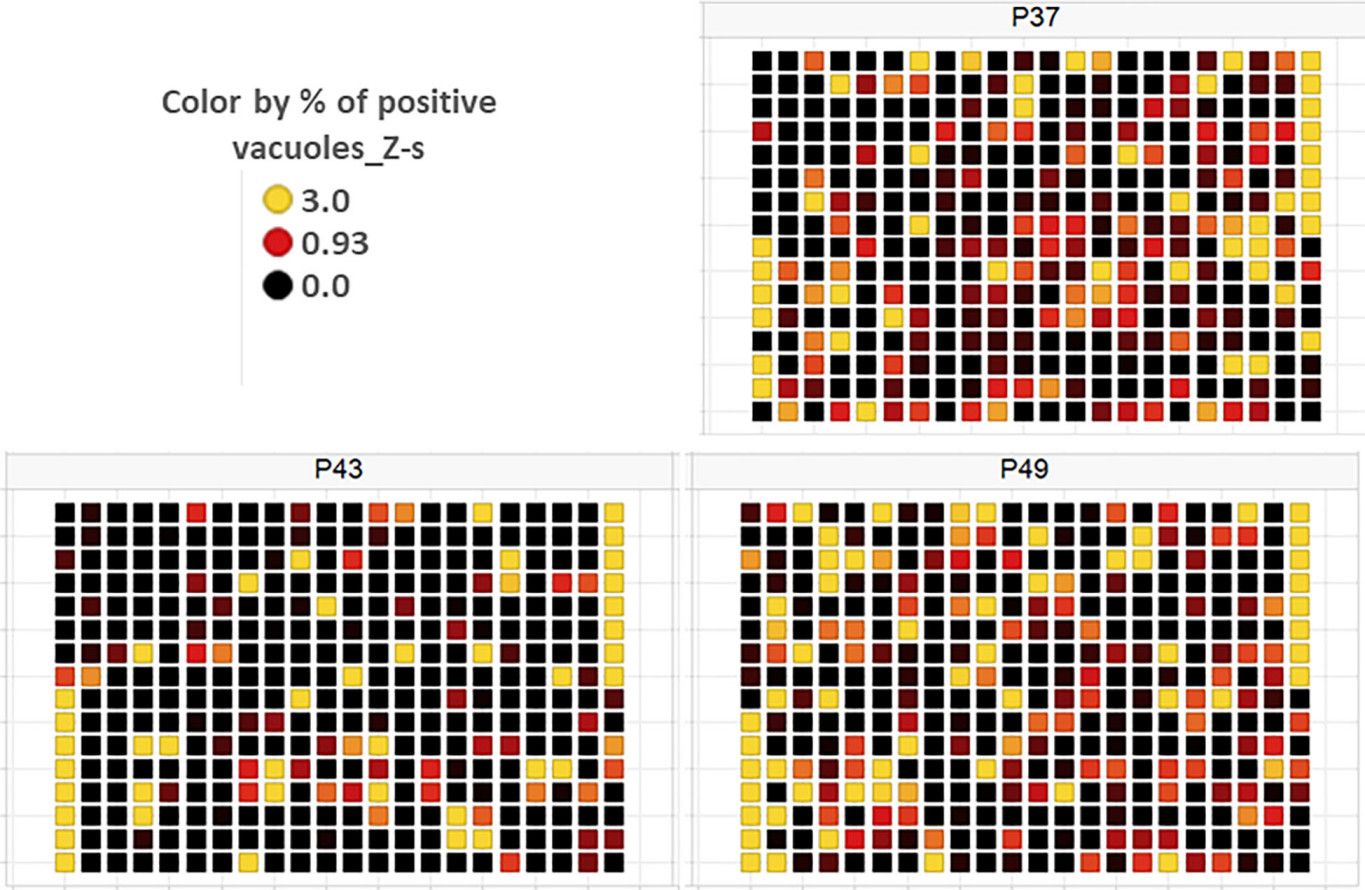
Plate heat map for each of the three screening plates (P37, P43 and P49) for each well, where each square corresponds to a well. DMSO was dispensed in the 1^st^ eight rows of the 1^st^ column and the last eight rows of the 21^st^ column, while the two compounds—SB 239063 (Cpd2) and SB 208 (Cpd3)—were placed in the last eight rows of the 1^st^ and the first eight rows of the 21^st^ column, respectively. For each well, the Z-score determined on the percentage of vacuoles displaying disruption is given, with Z-score > 3 indicating a compound impacting vacuole disruption.

**Figure 6:**
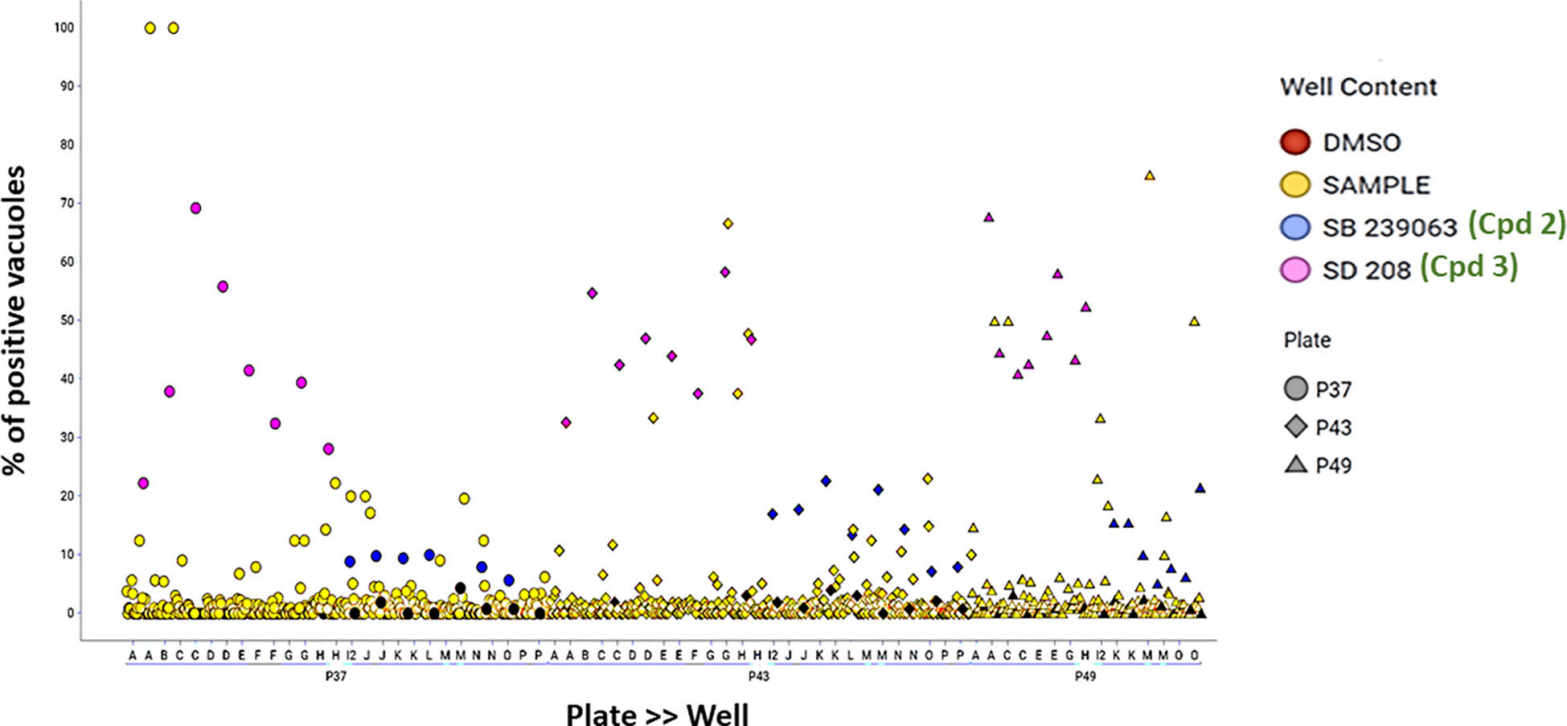
Data dot plot for each of the three screening plates—P37 (circles), P43 (diamonds) and P49 (triangles). The percentage of positive vacuoles is shown before normalization on the Y-axis. Red color corresponds to the negative control (DMSO), and blue and pink denote the positive controls—SB 239063 (Cpd2) and SB 208 (Cpd3)—in each plate, while yellow color is used for the tested compounds and black for non-infected wells.

**Figure 7:**
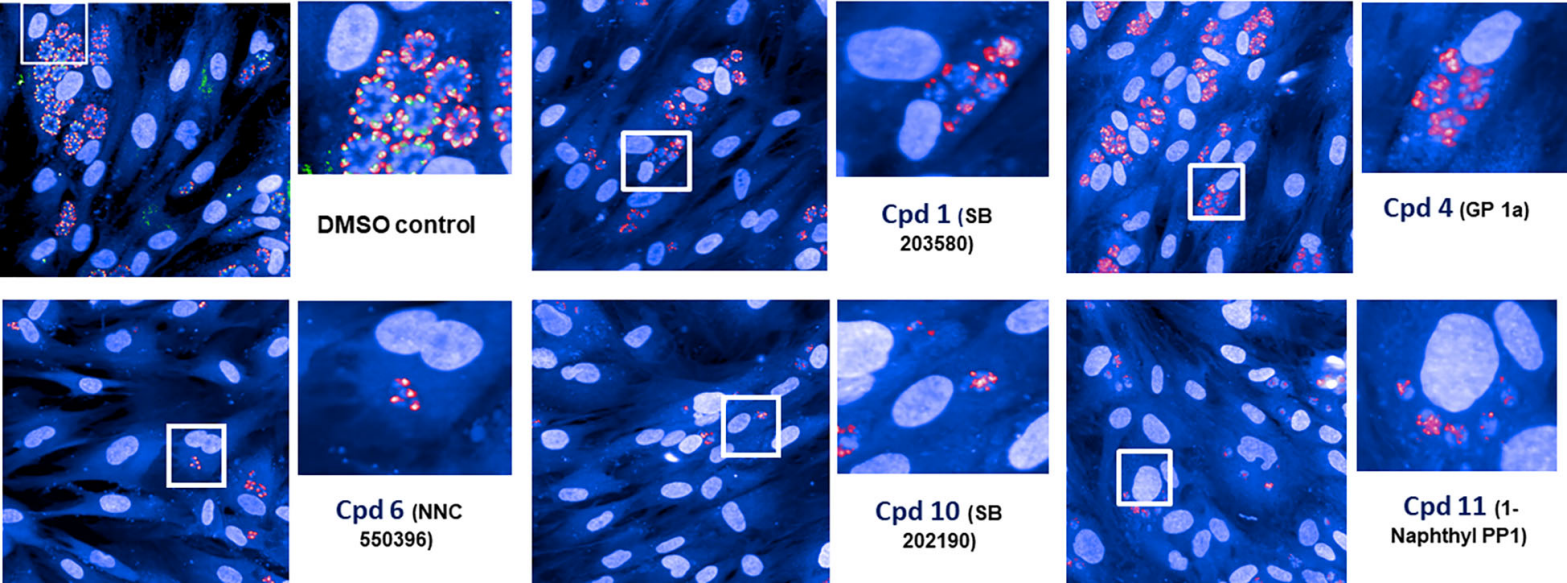
Screening hits. Five hits were obtained using our automatic image-based screening protocol and these fluorescence images (featuring necrotized intracellular parasites) illustrate the disruption of classical apical location of ROP1 signal compared to the negative DMSO control that shows intracellular dividing tachyzoites with normal morphology.

**Figure 8:**
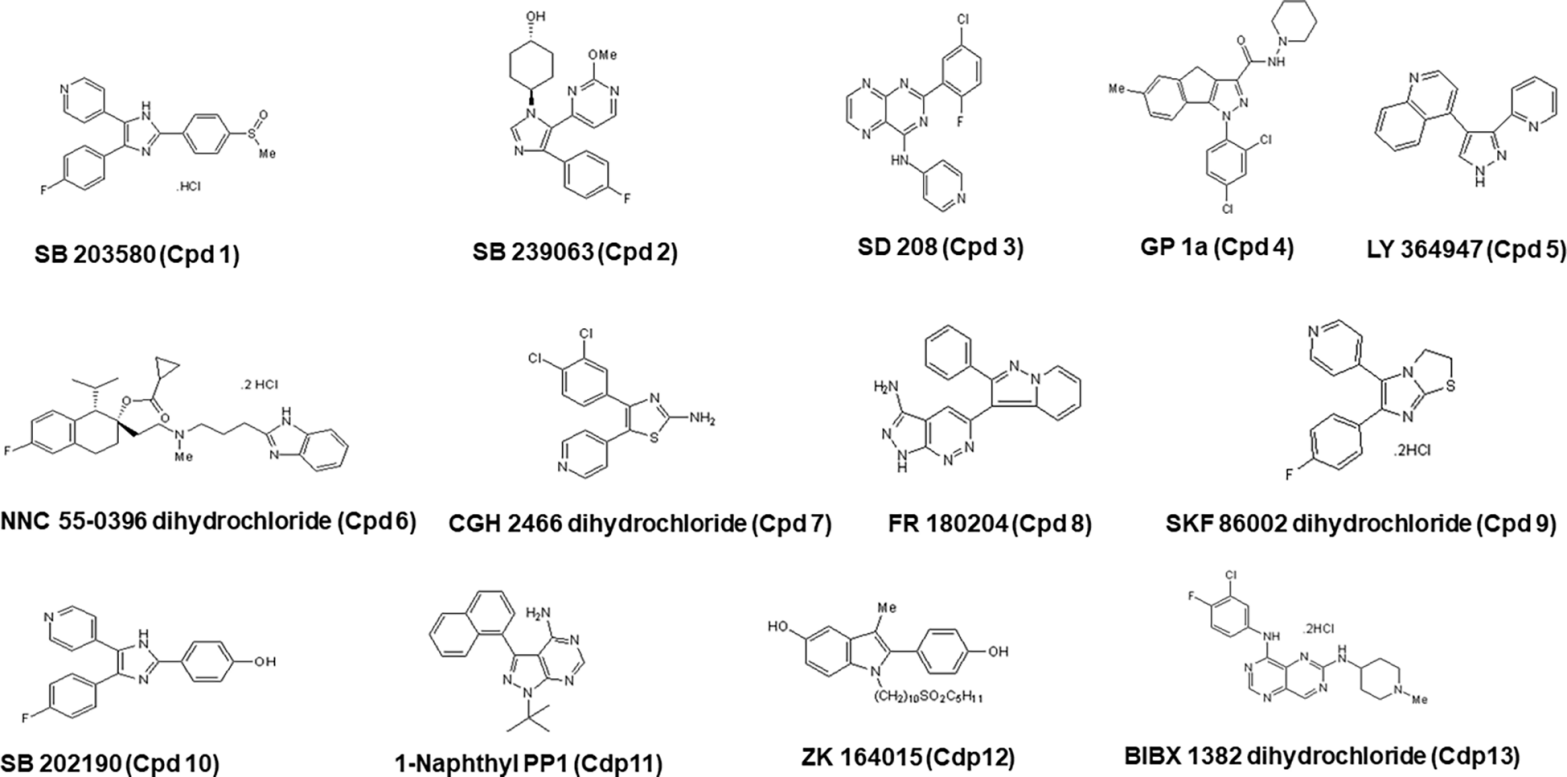
Chemical structures of all 12 hits with their corresponding names in the Tocris library, along with the nomenclature used in the present study (Cpd) and with the number corresponding to the order of identification.

As a part of the present study, a pre-screen optimization was also performed by filling two 384-well plates with two Pyr doses (0.6 µM and 10 µM) in a checkerboard style (**Figure 4A**). For the DMSO negative control, 20 parasites were noted on average per HFF cell, declining to about 2 parasites in cells containing 10 µM Pyr. Next, the first 384-well plate from the Tocris library in **Supplementary Table 2** was analyzed under the conditions described above. For this purpose, we generated a data processing script utilizing the signal emitted by the ROP1-mCherry and correlating it with the mislocalisation of the rhoptry signal. We first tested 300 compounds of Tocris library and the initial results pertaining to the wells corresponding to a compound yielding positive results were visually validated to concur with the findings related to the rhoptry marker mislocalisation. For the DMSO negative control, the rhoptry fluorescence signal was in the apical form of very distinct spots, while it was largely diffuse for the SB 239063 (Cpd2) and SB 208 (Cpd3) compounds, which correlated with a disruption of the classical and apical pattern of rhoptries (**Supplementary Figure 3**). Cpd2 and Cpd3 were identified as p38-MAP kinase and TGF-β type I receptor inhibitors, which were reported to reduce *T. gondii* growth by several authors (Wei et al., 2002; Wiley et al., 2010), indicating that our screen is robust. Thus, Cpd2 and Cpd3 were used as positive controls for the automatic screening of the 900 remaining small molecules distributed in three additional 384-well plates, as shown in **Figure 5**. During the analysis script creation, the dual GFP and mCherry labelling approaches were tested but it appears that ROP1-mCherry signal was sufficient to determine the effect of drugs on the parasite morphology inside the parasitophorous vacuole. Indeed, the TgSORT-GFP fluorescence was concentrated and created puncta, whereas the ROP1-mCherry created larger structured area in which the vacuole disruption can be captured in a more visible manner. Therefore, the percentage of positive vacuoles that correlate with the disruption of rhoptry signal was chosen as the key parameter, and the corresponding data plot is shown in **Figure 6**. These automatic image-based screenings identified nine further compounds that exhibited the expected phenotypic disruption fluorescence, as demonstrated in **Figure 7**. Unlike the negative DMSO control showing viable well-shaped intracellular tachyzoites with distinct apical red signal corresponding to normal rhoptries, these drug-treated wells contained necrotized intracellular parasites. When our visual and automatic screening methods were combined, 12 such compounds were identified, and their chemical structures are shown in **Figure 8**, revealing inhibitors of one CB_2_ receptor, two ATP-competitive TGF-βRI, one Ca_V_3.x pump and one estrogen receptor. The remaining seven compounds are kinase inhibitors, five of which inhibit p38-MAP kinase, and two inhibit ERK and Src family kinases, respectively (**Table 2**).

**Table 2:** Chemical names and known functions of compounds identified.

Compound	Chemical name	Function
**Cpd 1** (SB 203580)	4-[5-(4-Fluorophenyl)-2-[4-(methylsulphonyl)phenyl]-1*H*-imidazol-4-yl]pyridinehydrochloride	Selective inhibitor of p38 MAPK
**Cpd 2** (SB 239063)	*trans*-4-[4-(4-Fluorophenyl)-5-(2-methoxy-4-pyrimidinyl)-1*H*-imidazol-1-yl]cyclohexanol	Potent, selective p38 MAPK inhibitor
**Cpd 3** (SD 208)	2-(5-Chloro-2-fluorophenyl)-4-[(4-pyridyl)amino]pteridine	Potent ATP-competitive TGF-BRI inhibitor
**Cpd 4** (GP 1a)	*N*-(Piperidin-1-yl)-1-(2,4-dichlorophenyl)-1,4-dihydro-6-methylindeno[1,2-*c*]pyrazole-3-carboxamide CB2	CB_2_ receptor inverse agonist
**Cpd 5** (LY 364947)	4-[3-(2-Pyridinyl)-1*H*-pyrazol-4-yl]-quinoline	Selective inhibitor of TGF-βRI
**Cpd 6** (NNC 55-0396 dihydrochloride)	(1*S*,2*S*)-2-[2-[[3-(1*H*-Benzimidazol-2-yl)propyl]methylamino]ethyl]-6-fluoro-1,2,3,4- Retrahydro-1-(1-methylethyl)-2-naphthalenylcyclopropanecarboxylate dihydrochloride	Highly selective Ca_v_3.x blocker
**Cpd 7** (CGH 2466 dihydrochloride)	4-(3,4-Dichlorophenyl)-5-(4-pyridinyl)-2-thiazolamine dihydrochloride	A_1_, A_2B_ and A_3_ antagonist and inhibitor of p38 MAPK and PDE4
**Cpd 8** (FR 180204)	5-(2-Phenyl-pyrazolo[1,5-a]pyridin-3-yl)-1*H*-pyrazolo[3,4-*c*]pyridazin-3-ylamine	Selective ERK inhibitor
**Cpd 9** (SKF 86002 dihydrochloride)	6-(4-Fluorophenyl)-2,3-dihydro-5-(4-pyridinyl)imidazo[2,1-*b*]thiazoledihydrochloride	p38 MAPK inhibitor; anti-inflammatory agent
**Cpd 10** (SB 202190)	4-[4-(4-Fluorophenyl)-5-(4-pyridinyl)-1*H*-imidazol-2-yl]phenol	Potent, selective inhibitor of p38 MAPK
**Cpd 11** (1-Naphthyl PP1)	1-(1,1-Dimethylethyl)-3-(1-naphthalenyl)-1*H*-pyrazolo[3,4-*d*]pyrimidin-4-amine	Src family kinase inhibitor; also inhibits c-Abl
**Cdp 12** (ZK 164015)	2-(4-Hydroxyphenyl)-3-methyl-1-[10-(pentylsulfonyl)decyl]-1*H*-indol-5-ol	Potent estrogen receptor antagonist

### Dose-dependent inhibitory effects of the small molecules on *T gondii* growth

**Figure 9:**
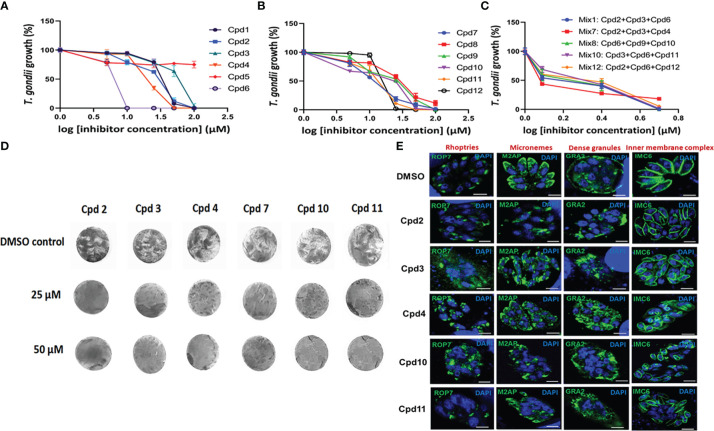
Dose-dependent inhibitory activities on the 12 compounds using β-galactosidase assays. Panel **(A)** shows the assays performed on the first six compounds (including Cpd5, which probably represents a false positive). Panel **(B)** illustrates the six remaining compounds. These two panels correspond to single-dose assays and all p values are below 0.0001. Panel **(C)** pertains to a mix of three compounds (p value = 0.0024), while Panel **(D)** shows a dose-dependent inhibition of plaque formation by *T. gondii* after nine days of drug exposure. As two independent experiments yielded identical results, one is illustrated here. Panel **(E)** showing confocal microscopy of intracellular tachyzoites treated with 25 µM of drugs indicated on the images for 24h followed by staining with different antibodies specific to rhoptries, micronemes, dense granules and the inner complex membrane. The control corresponds to intracellular parasites treated with DMSO alone. Nuclei are stained with DAPI. Bar, 3 µm.

The inhibitory activity of the compounds identified through image-based screening was validated by another enzymatic method and the findings revealed a highly significant difference (p < 0.0001) in parasite growth depending on the inhibitor presence and concentration used, reflecting an important effect of inhibitors on the intracellular replication of *T. gondii* using β-galactosidase assays (**Figures 9A, B**). As compound Cpd5 failed to produce inhibitory effect (**Figure 9A**), it was excluded from the validation process. When the positive compounds were placed in groups of three, their joint IC_50_ decreased from 15−60 µM to 1.5−2.5 µM. The concentrations below the IC_50_ of each individual compound was mostly chosen and these compounds were combined according to their ability to inhibit different putative targets or pathways (**Figure 9C**). Each compound of the combined drug mixtures was used at a range of concentrations between 1.0 to 3.0 µM. Nonetheless, the difference observed between the results obtained by applying inhibitor mixes to *T. gondii* and the untreated parasites was significant (p = 0.0024), indicating beneficial synergistic effect. Moreover, when infected confluent HFF cells were subjected to drug inhibition for nine days to allow plaque formation, which corresponds to several rounds of invasion, egress and reinvasion, parasite growth was completely abolished for all six compounds tested, i.e., Cpd2, Cpd3, Cpd4, Cpd7, Cpd10 and Cpd11 (**Figure 9D**). Even at high concentrations (25 and 50 µM), the confluent monolayer of HFF cells remained intact, as shown by homogenous crystal violet staining at these doses. Furthermore, we determined the effects of these drugs on the classical fluorescence patterns of few intracellular organelles of the parasites using specific antibodies and confocal imaging. **Figure 9E** showed that intracellular tachyzoites of parental RH strain treated with Cpd2, 3, 4, 10 and 11 led to detrimental effects on the apical signal of rhoptries with from more fragmented patterns until a total disappearance of the fluorescence signals in some parasites. These drugs also severely affected the apical pattern of micronemes except for Cpd 6 as compared to the four other drugs. The compounds Cpd2 and Cpd10 were less active on the pattern of secretion dense granule proteins into parasitophorous vacuoles (**Figure 9E**). The inner complex membranes of drug-treated parasites appears normal, except that the parasites were shorter in sizes compared to parasites treated with DMSO alone (**Figure 9E**). As expected, these parasites treated with DMSO alone showed typical fluorescence signals in the apical end for rhoptries and micronemes. Altogether, these data confirms the detrimental effects observed on ROP1-mCherry containing rhoptries in the intracellular parasites during our automatic and miniaturized drug screening.

### Effect of the identified compounds on *P. falciparum*

**Figure 10:**
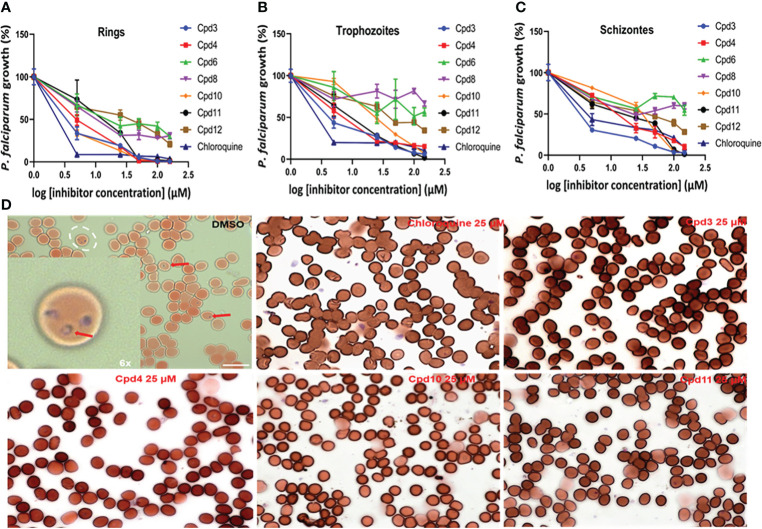
Dose-dependent inhibitory effects of the studied drugs on *P. falciparum* growth in red blood cells (RBCs). Panels **(A–C)** respectively showing ring stage, trophozoite and schizonte counts after drug treatments based on flow cytometry, with chloroquine as positive control (p < 0.0001). Panel **(D)** shows a blood smear used to visualize ring stages of the DMSO control versus selected drugs in all used concentrations.

To test the inhibitory effects of eight compounds identified during our image-based screen on *P. falciparum* growth, we used infected red blood cells (RBCs) synchronized twice with sorbitol and subjected them to flow cytometry analysis. The findings indicated that four compounds (Cpd3, Cpd4, Cpd10 and Cpd11) were active against *P falciparum* and the infected RBC cultures were cleared of ring stages (**Figure 10A**). We estimated the IC_50_ of these drugs between 1.0 to 5.0 µM. Moreover, increasing inhibitor concentrations resulted in a significant (p < 0.0001) decrease in *P. falciparum* growth in RBCs. When trophozoite and schizonte forms of *P falciparum* were counted by flow cytometry (**Figures 10B, C**), identical inhibitory effect of these four compounds was affirmed. The remaining four compounds inhibited *P falciparum* growth, but their activity was below that noted for Cpd3, Cpd4, Cpd10 and Cpd11 (**Figure 10**). In all cases, inhibitory activity and absence of ring stage in the infected RBCs were confirmed by direct observation of stained red blood smears, as shown in **Figure 10D**. Only the negative DMSO controls exhibited numerous ring stages, confirming that these compounds are capable of directly targeting parasites, since *P. falciparum* was grown in non-nucleated red blood cells characterized by reduced metabolism and lack of intracellular organelles.

## Discussion

*T. gondii* and *P. falciparum* share evolutionary history, as both contain orthologous proteins often associated with similar cellular processes and likely exhibit similar sensitivity to inhibitors that affect their unique life stage events. Guided by these findings, we developed a powerful and efficient image-based screening protocol involving transgenic *T. gondii* simultaneously expressing TgSORT-GFP (green signal) and ROP1-mCherry (red signal) at two distinct subcellular locations, which can be applied to the extant library of 1,120 compounds. We also found that the signal of ROP1-mCherry alone is accurate and efficient for our automatic miniaturized drug screening. Our aim was to identify drugs capable of disrupting subcellular localization of fluorescence signals, thereby compromising parasite’s polarity, morphology and intracellular replication. For this purpose, we employed experimental conditions that would allow us to identify compounds that not only inhibit but also disrupt parasite growth and its classical apical signal of rhoptries. Our findings revealed that, in addition to the DAPI signal enabling detection of both HFF and parasite nuclei, the red signal was sufficient to identify the disruption of the apically located fluorescence of rhoptries during the screening process. This protocol was thus adopted in automated image analysis, which resulted in the identification of seven kinase inhibitors that mostly inhibited p38-MAP kinase, and two of which—Cpd2 (SB 203580) and Cpd3 (SB 208)—were previously reported to reduce *T. gondii* growth (Wei et al., 2002; Wiley et al., 2010). It should be emphasized that the compound library used in our analyses was used by Dittmar et al. (2016) to assess inhibition of *T. gondii* growth based on β-galactosidase assay and these authors identified 94 compounds and a significant number of small molecules known to impact dopamine or estrogen signaling. Among the 12 compounds identified in our screen, four inhibitors (Cpd4, Cpd6, Cpd9 and Cpd12) were not present in the hits reported by Dittmar et al. (2016). In addition, one of the compounds identified in our study (Cpd12, ZK 164015) as an estrogen receptor antagonist has not been identified in the aforementioned screening. These data suggest that even the same library was used; different hits can be obtained according to the methodology developed for the drug screening. It is also worth noting that Touquet et al. (2018) developed an automated image-based strategy for screening a library of compounds belonging to four classes of either natural compounds or synthetic derivatives. Next, we established the kinetics of inhibition of *T. gondii* proliferation by the identified 12 active compounds using enzymatic assay based on the β-galactosidase activity. We further confirmed that some of these compounds are also capable of inhibiting or eliminating *P falciparum* grown in red blood cells. Interestingly, four of these compounds (potent ATP-competitive TGF-βRI inhibitor, CB_2_ receptor inverse agonist, p38-MAPK and Src family kinase inhibitor) were at micromolar potency, as established using flow cytometry method. These observations suggest that the effects of these compounds are efficiently harmful to the intracellular growth of *T. gondii*. It is interesting to note that our screen revealed several kinase inhibitors, which is relevant given that both *P. falciparum* and *T. gondii* contain a large number of proteins that are phosphorylated through action of several kinases, including tyrosine kinases (Treeck et al., 2011). The secreted proteins included an expanded, lineage-specific family of protein kinases termed rhoptry kinases (ROPKs), several of which have been shown to be key virulence factors. For example, ROP16 targets the nucleus and phosphorylates STAT3/6, and regulates the immune responses (Saeij et al., 2006; Taylor et al., 2006; Saeij et al., 2007; Yamamoto et al., 2009; Ong et al., 2010; Butcher et al., 2011). Thus, it is likely that the kinase inhibitors identified during our screen may have some of these parasite kinases as targets. Consequently, our approaches may be useful in the future for repurposing drugs with known safety profiles that could present attractive and significant advantages in terms of anti-parasite drug development. Although p38-MAP kinase inhibitors appear to be toxic, as indicated by adverse effects during previous clinical trials (Patnaik et al., 2016), other drugs (unrelated to kinase inhibitors) identified as a part of the present study can be considered. Based on the obtained findings, we postulate that a combination of drugs acting *via* multiple effector mechanisms might be superior to a single drug or a group of drugs with the same mechanism of action, because developing parasite resistance against multiple effector mechanisms simultaneously would be extremely hard to achieve. Further work on target identification and mechanism analysis is thus required to facilitate the development of anti-parasitic compounds with cross-species efficacy. Establishing the linkages between unique chemical scaffolds and their resultant cellular phenotypes on two evolutionarily related yet distinct parasites will provide an avenue for conducting detailed mechanistic studies with the organism of choice. In particular, biochemical and/or genetic approaches should be employed to identify the drug targets for further design of new parasite-specific inhibitors.

## Data availability statement

The original contributions presented in the study are included in the article/**Supplementary Material**. Further inquiries can be directed to the corresponding author.

## Author contributions

The author(s) have made the following declarations about their contributions: Conceived and designed the experiments: PB and ST. Performed the experiments: AH, RH, and AV. Contributed reagents/materials/analysis tools: SD, ON, and CD. Performed data analysis: PB and AV. Wrote the paper: AH, PB, and ST. All authors contributed to the article and approved the submitted version.

## Supplementary material

Supplementary Figure 1 Acoustic dispensing principles and apparatus.

Supplementary Figure 2 Pre-screening of effective pyrimethamine and DMSO concentrations allowing the image-based drug identification. **(A)** Impact of different DMSO concentrations on the uninfected HFF cells. **(B)**: Two independent experiments using different pyrimethamine concentrations tested on 7,000 infected HFF cells. **(C)** Dose-dependence of pyrimethamine concentrations based on tests involving 7,000 and 13,000 infected HFF cells. **(D)** Determination of the pyrimethamine EC_50_ under our experimental conditions designed for drug screening.

Supplementary Figure 3 First screening hits of the 380-well plate containing 200 small molecules based on direct and manual microscopic observations. Two compounds—Cpd2 (SB 239063) and Cpd3 (SD 208)—were identified.

Supplementary Figure 4 Testing drug toxicity on uninfected and infected HFF cells. **(A)** Cpd1 (SB 203580) at 40 µM; **(B)** Cpd1 (SB 203580) at 5 µM; **(C)** Cpd2 (SB 239063) at 40 µM; **(D)** (SB 239063) at 5 µM; **(D)** Cpd3 (SD 208) at 40µM, **(E)** Cpd3 (SD 208) at 5 µM. **(G)** DMSO; **(H)** No treatment. **(I)** Test of toxicity on uninfected HFF cells using different Cpd1, Cpd2 and Cpd3 concentrations, revealing absence of toxicity. **(J)** Number of HFF cells containing positive vacuoles across the dose-dependent inhibitory activity ranges of these three drugs. The number of positive vacuoles has not significantly changed.

Supplementary Table 1 Columbus image analysis script.

Supplementary Table 2 Small molecules, their corresponding chemical structures and functions based on the Tocris library data.

Supplementary Table 3 Four drugs (30%) among the twelve inhibitors identified during our screening are not present in those reported by Ditmer et al. (2016).
