# Supplementary figures and images for: An image-based high-content screening for compounds targeting Toxoplasma gondii repurposed inhibitors effective against the malaria parasite Plasmodium falciparum
Source: Front Cell Infect Microbiol. 2023 Mar 3;13:1102551. doi: 10.3389/fcimb.2023.1102551 (PMC10020723; doi:10.3389/fcimb.2023.1102551)

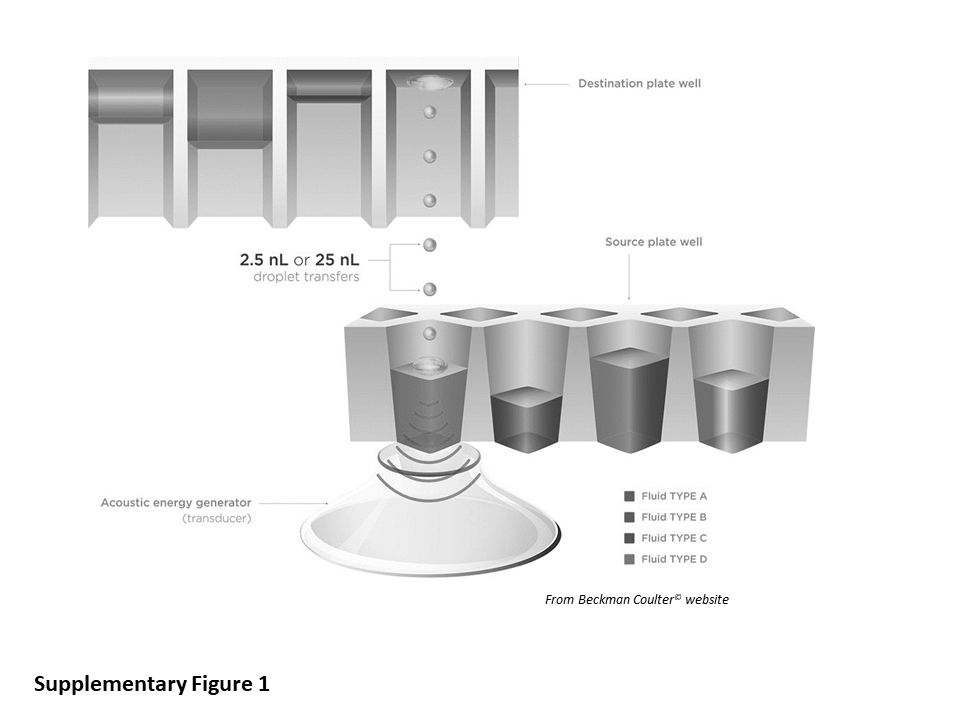

Supplement: Supplementary Figure 1 — Acoustic dispensing principles and apparatus. [file Image_1.tif]

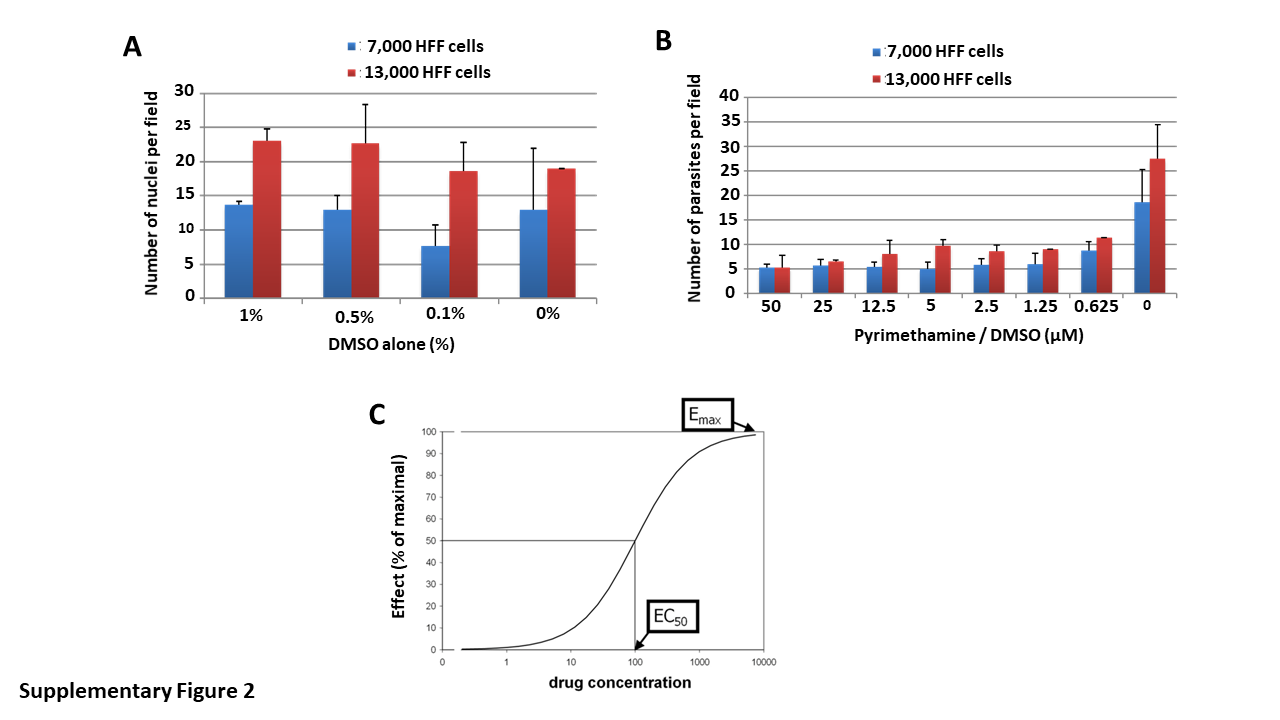

Supplement: Supplementary Figure 2 — Pre-screening of effective pyrimethamine and DMSO concentrations allowing the image-based drug identification. (A) Impact of different DMSO concentrations on the uninfected HFF cells. (B): Two independent experiments using different pyrimethamine concentrations tested on 7,000 infected HFF cells. (C) Dose-dependence of pyrimethamine concentrations based on tests involving 7,000 and 13,000 infected HFF cells. (D) Determination of the pyrimethamine EC50 under our experimental conditions designed for drug screening. [file Image_2.tif]

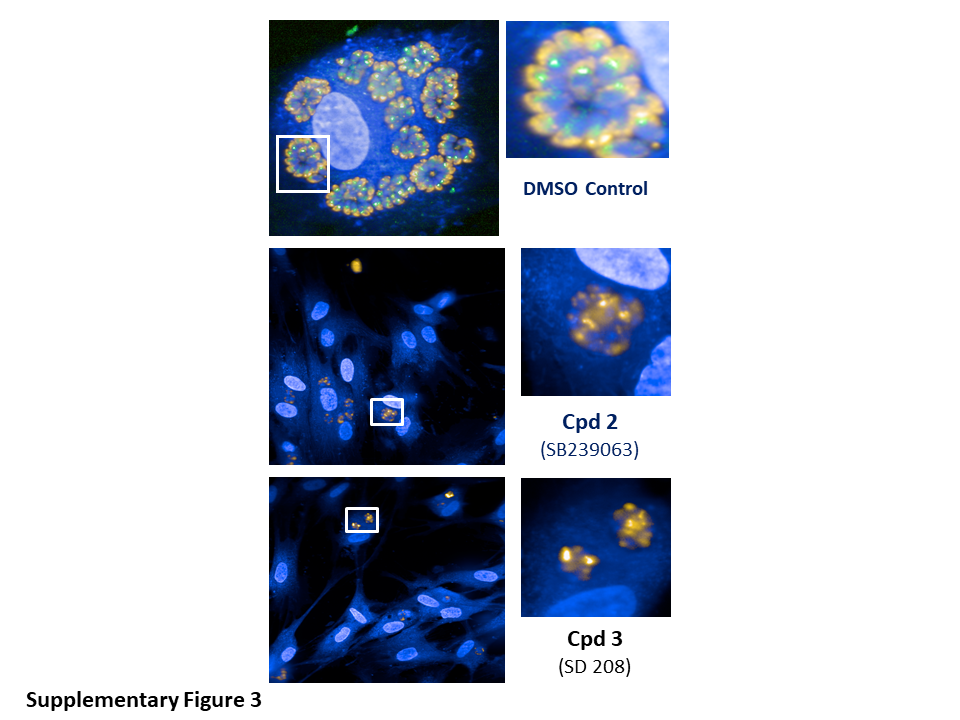

Supplement: Supplementary Figure 3 — First screening hits of the 380-well plate containing 200 small molecules based on direct and manual microscopic observations. Two compounds—Cpd2 (SB 239063) and Cpd3 (SD 208)—were identified. [file Image_3.tif]

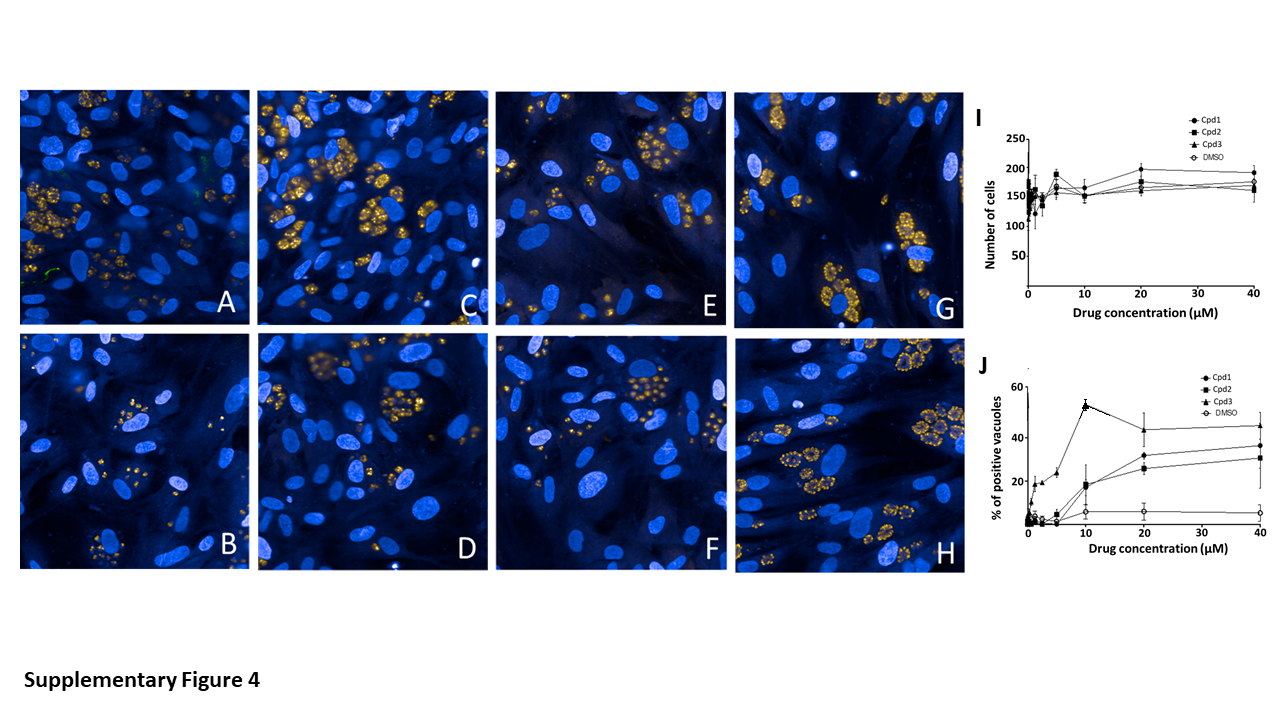

Supplement: Supplementary Figure 4 — Testing drug toxicity on uninfected and infected HFF cells. (A) Cpd1 (SB 203580) at 40 µM; (B) Cpd1 (SB 203580) at 5 µM; (C) Cpd2 (SB 239063) at 40 µM; (D) (SB 239063) at 5 µM; (D) Cpd3 (SD 208) at 40µM, (E) Cpd3 (SD 208) at 5 µM. (G) DMSO; (H) No treatment. (I) Test of toxicity on uninfected HFF cells using different Cpd1, Cpd2 and Cpd3 concentrations, revealing absence of toxicity. (J) Number of HFF cells containing positive vacuoles across the dose-dependent inhibitory activity ranges of these three drugs. The number of positive vacuoles has not significantly changed. [file Image_4.tif]

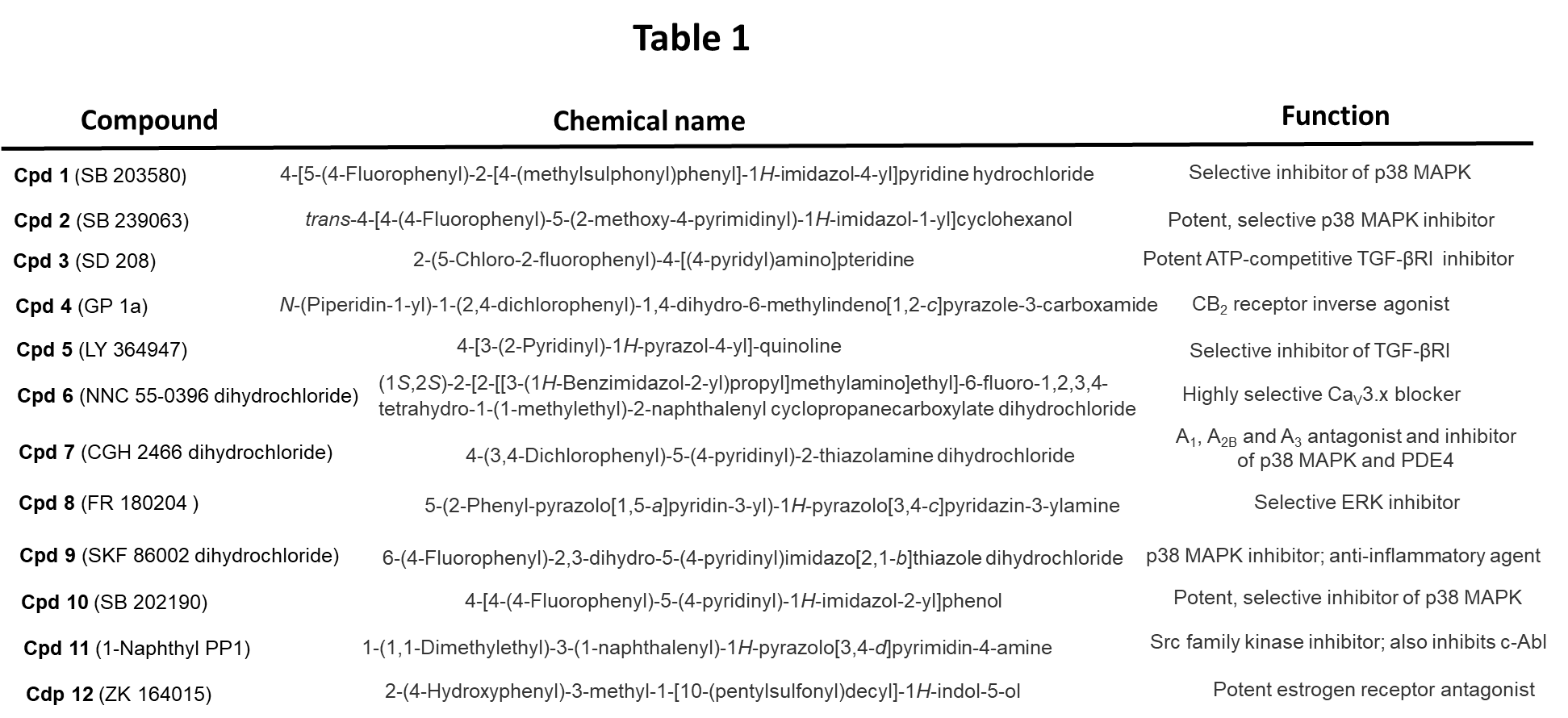

Supplement: Supplementary Table 1 — Columbus image analysis script. [file Table_1.docx]
