# Supplementary material for: An image-based high-content screening for compounds targeting Toxoplasma gondii repurposed inhibitors effective against the malaria parasite Plasmodium falciparum
Source: Front Cell Infect Microbiol. 2023 Mar 3;13:1102551. doi: 10.3389/fcimb.2023.1102551 (PMC10020723; doi:10.3389/fcimb.2023.1102551)
Supplement: Supplementary Table 3 — Four drugs (30%) among the twelve inhibitors identified during our screening are not present in those reported by Ditmer et al. (2016). [file Table_3.doc]

| **Our Data** | | | | | **Ditmar Data** | |
| --- | --- | --- | --- | --- | --- | --- |
| Cpd Number | Number of Cells | % Positive Vacuoles Normalized | Plate | Well | IC50 | Average Cell Viability |
| 1 | 124 | 17.13 | 37 | M05 | 2.1-2.4 | 89.42 |
| 2 | 155 | 1.3 | 31 | H11 | 0.2-0.3 | 93.54 |
| 3 | 62 | 2.55 | 31 | G06 | 0,3-0,4 | 82.9 |
| 4 | 158 | 0.08 | 49 | K05 | NA | NA |
| 5 | 156 | 17.94 | 49 | I05 | 1.6-2.1 | 85.08 |
| 6 | 127 | -0.51 | 37 | N17 | NA | NA |
| 7 | 171 | 1.37 | 37 | H12 | 1.0-1.3 | 87.41 |
| 8 | 91 | 10.7 | 37 | G04 | 1.9-5.1 | 94.68 |
| 9 | 163 | 5.6 | 37 | E16 | NA | NA |
| 10 | 167 | 14.86 | 37 | J15 | 0.7-0.8 | 91.51 |
| 11 | 194 | 45.32 | 43 | H21 | 0.4-0.8 | 83.62 |
| 12 | 181 | 17.43 | 37 | I20 | NA | NA |

NA : Not active

Supplementary Table 3
